# UGT2B17 modifies drug response in chronic lymphocytic leukaemia

**DOI:** 10.1038/s41416-020-0887-6

**Published:** 2020-05-18

**Authors:** Eric P. Allain, Michèle Rouleau, Katrina Vanura, Sophie Tremblay, Joanie Vaillancourt, Vincent Bat, Patrick Caron, Lyne Villeneuve, Adrien Labriet, Véronique Turcotte, Trang Le, Medhat Shehata, Susanne Schnabl, Dita Demirtas, Rainer Hubmann, Charles Joly-Beauparlant, Arnaud Droit, Ulrich Jäger, Philipp B. Staber, Eric Lévesque, Chantal Guillemette

**Affiliations:** 1 Pharmacogenomics Laboratory, Centre Hospitalier Universitaire de Québec (CHU de Québec) Research Center and Faculty of Pharmacy, Laval University, Québec, QC Canada; 2 Division of Hematology and Hemostaseology, Department of Medicine I and Comprehensive Cancer Center, Medical University of Vienna, Vienna, Austria; 3 CHU de Québec Research Center and Department of Molecular Medicine, Faculty of Medicine, Laval University, Québec, QC Canada; 4 CHU de Québec Research Centre, Department of Medicine, Faculty of Medicine, Laval University, Québec, QC Canada; 5 Canada Research Chair in Pharmacogenomics, Québec, QC Canada

**Keywords:** Oncology, Predictive markers, Haematological cancer

## Abstract

**Background:**

High UGT2B17 is associated with poor prognosis in untreated chronic lymphocytic leukaemia (CLL) patients and its expression is induced in non-responders to fludarabine-containing regimens. We examined whether UGT2B17, the predominant lymphoid glucuronosyltransferase, affects leukaemic drug response and is involved in the metabolic inactivation of anti-leukaemic agents.

**Methods:**

Functional enzymatic assays and patients’ plasma samples were analysed by mass-spectrometry to evaluate drug inactivation by UGT2B17. Cytotoxicity assays and RNA sequencing were used to assess drug response and transcriptome changes associated with high UGT2B17 levels.

**Results:**

High UGT2B17 in B-cell models led to reduced sensitivity to fludarabine, ibrutinib and idelalisib. UGT2B17 expression in leukaemic cells involved a non-canonical promoter and was induced by short-term treatment with these anti-leukaemics. Glucuronides of both fludarabine and ibrutinib were detected in CLL patients on respective treatment, however UGT2B17 conjugated fludarabine but not ibrutinib. AMP-activated protein kinase emerges as a pathway associated with high UGT2B17 in fludarabine-treated patients and drug-treated cell models. The expression changes linked to UGT2B17 exposed nuclear factor kappa B as a key regulatory hub.

**Conclusions:**

Data imply that UGT2B17 represents a mechanism altering drug response in CLL through direct inactivation but would also involve additional mechanisms for drugs not inactivated by UGT2B17.

## Background

Chronic lymphocytic leukaemia (CLL) is the most frequent adult leukaemia in the western world. Cancer growth varies widely with indolent and aggressive forms of CLL. In recent years, there has been substantial progress in the clinical management of CLL patients supported by a better risk stratification and the introduction of a number of novel therapeutic agents.^1^ These advances significantly improved clinical outcomes of CLL patients.

Pre-treatment evaluation of informative prognostic markers helps the stratification of CLL patients into risk subgroups. These markers include unmutated immunoglobulin heavy chain variable region (U-IGHV), del(17p) or *TP53* mutations, which are associated with poor response to treatment and predict earlier relapse after achieving initial haematological remission.^2^ For treatment-naïve patients with these high-risk features, the Bruton tyrosine kinase (BTK) inhibitor ibrutinib is indicated as an initial systemic therapy rather than chemoimmunotherapy.^3,4^ The latter is based on the purine analogue fludarabine or alkylating agent bendamustine or chlorambucil backbones combined to the monoclonal antibody rituximab directed against the CD20 B-cell marker.^5^ Other therapeutic small molecules include two distinct mechanisms of actions with venetoclax, a BCL-2 inhibitor,^6^ idelalisib, a phosphatidylinositol-3-kinase (PI3K) δ inhibitor, and duvelisib, a dual inhibitor of PI3K δ and γ isoforms.^7,8^ Phase 3 trials are ongoing for additional agents such as the second-generation oral BTK inhibitor acalabrutinib and the Syk/Jak inhibitor cerdulatinib.^9,10^ Whilst treatment has significantly advanced patients’ survival, it is also associated with incomplete clonal eradication, relapse, refractoriness or transformation to a more aggressive lymphoma (Richter’s syndrome).^3^

Both innate and acquired resistance represent major challenges for long-term disease control and an intense area of research. Findings reveal that targeted agents have unique mechanisms of resistance compared with chemotherapy. Genetic anomalies associated with fludarabine refractoriness include mutations in *TP53, SF3B1, NOTCH1* and *BIRC3* genes.^11,12^ Other genetic screens have recently identified further genes that could be implicated in fludarabine sensitivity such as *ARID5B* and *BRAF*.^13,14^ As targeted therapy agents have become more established in cancer therapy, recent reports attribute acquired hypermorphic mutations in genes of targeted pathways, including BTK and its immediate downstream effector phospholipase C, γ2 (PLCG2) critical for BCR signalling.^15^ Mechanisms of resistance to other targeted agents, so early in clinical use, have not yet been portrayed in CLL patients but work is ongoing in this area.

Additional contributing mechanisms of reduced drug sensitivity that may be shared by targeted agents and chemotherapy may include increased drug efflux by ATP-dependent transporters and drug inactivation by metabolising pathways such as the glucuronidation pathway by UDP-glucuronosyltransferase enzymes (UGTs). By conjugating lipophilic drugs to glucuronic acid (GlcA), UGTs impair biological activity and enhance water solubility of drugs, driving their elimination and a lack of drug efficacy.^16^ Accumulating evidence support an influence of the UGT pathway to drug resistance in many cancers including in leukaemias, namely in CLL patients treated with fludarabine and acute myeloid leukaemia (AML) patients receiving ribavirin or cytarabine-based treatments.^17–19^ However, for most drugs used in CLL, the glucuronidation pathway has not been comprehensively studied. Reports have established that high UGT2B17 expression is an adverse prognostic factor in CLL, associated with shorter treatment-free survival, overall survival and patients requiring more treatment.^17,20,21^ The link between UGT2B17, adverse CLL features and progression appears complex, and precise molecular mechanisms underlying these observations remain to be elucidated. In cohorts of CLL patients, UGT2B17 was associated with unmutated IgHV and predicted poor survival,^17^ and was further associated with progressive disease within the group of patients with a more favourable profile (mutated IgHV).^21^ A recent study supports that UGT2B17-mediated metabolism would participate in the regulation of signalling pathways critical for CLL progression.^22^ High UGT2B17 expression in leukaemic cells was further linked to a lack of response to fludarabine-containing regimens.^17^ UGT2B17 is one of the 19 functional human drug-conjugating UGT enzymes, also involved in regulating homoeostasis of a number of endogenous metabolites, however its role in conjugating anti-leukaemics remains largely unknown.^16^

In the present study, we investigated the response to anti-leukaemic agents namely fludarabine, ibrutinib, idelalisib, bendamustine, chlorambucil, venetoclax, acalabrutinib, cerdulatinib and duvelisib in relation to UGT2B17 expression levels. We then sought to evaluate the UGT-mediated enzymatic inactivation of drugs relevant to CLL and the possible involvement of the UGT2B17 enzyme. Our data support that high UGT2B17 expression alters drug response by direct inactivation but likely also through other mechanisms. We further evidence that the UGT pathway is involved in the inactivation of the majority of anti-leukaemic agents used in CLL, suggesting that this pathway may be associated with drug resistance.

## Methods

### Patients, primary CLL cells and cell lines

Cryopreserved peripheral blood mononuclear cells (PBMCs) and plasma samples were obtained from CLL patients who were diagnosed between 1987 and 2011 at Vienna General Hospital. Patients provided informed consent. The study was carried out in accordance with the Declaration of Helsinki and was approved by the local ethical research committees of the Medical University of Vienna (Ethics vote 1499/2015) and the CHU de Québec (A14-10-1205). *UGT2B17* mRNA analysis was performed in CD19^+^-sorted cells of twenty CLL patients before and after the first cycle of treatment with fludarabine-containing regimens as described.^23^ Plasma samples for quantification of glucuronides in circulation were available for two patients in the 1st week after the first cycle of treatment with fludarabine-chlorambucil-rituximab and for 15 patients during treatment with ibrutinib. The B lymphoblastoid leukaemic cell lines included p53^wild-type^ (EHEB, JVM-2) and p53^mutated^ (MEC-1) cell lines purchased from ATCC (Manassas, VA, USA) or DSMZ (Braunschweig, Germany). Cells overexpressing UGT2B17 were previously described.^22^ Cell culture components were all purchased from Wisent Bioproducts (Saint-Bruno, QC, Canada).

### Drug conjugation assays

Glucuronidation assays were conducted using human liver, intestine and kidney microsomes (Xenotech, Kansas City, KS, USA) or supersomes expressing individual human UGT isoforms (Corning, MA, USA). In the absence of commercially available UGT2B11 supersomes, microsomal proteins derived from HEK293 expressing the recombinant UGT2B11 enzyme were used. Substrates included dihydrotestosterone (DHT) purchased from Steraloids (Newport, RI, USA), fludarabine and chlorambucil from Sigma-Aldrich (Oakville, ON, Canada) and bendamustine from Toronto Research Chemicals (North York, ON, Canada). All other compounds were purchased from Selleckchem (Houston, TX, USA). UGT assays were incubated at 37 °C for 6 h in a final volume of 100 µL of assay buffer containing 20 µg of proteins, 0.5 mM DTT, 10 mM MgCl_2_, 50 mM Tris-HCl (pH 7.5), 20 µg/mL alamethicin, 2 mM UDP-GlcA and 0.5 µg/mL leupeptin with the indicated substrate. Samples were then stopped by adding 100 µL cold methanol, centrifuged at 16,000×*g* for 10 min and supernatants analysed by mass spectrometry. The following negative controls were included for each analysis: reaction assays without substrate, without the microsomal fraction and without substrate and microsomal fraction.

### Mass spectrometry-based analysis of drug conjugation

The establishment of these analytical methods required the optimisation of chromatographic conditions, production of glucuronide standards using pooled liver microsomes and the use of deuterated molecules as internal standards (Clearsynth, Mississauga, ON, Canada). Drugs and their G metabolites were measured on an API 6500 mass spectrometer (Sciex, Concord, ON, Canada), operated in multiple reactions monitoring mode and equipped with a turbo ion-spray source. Electrospray ionisation was performed in the positive ion mode. The chromatographic system consisted of a Nexera (Shimadzu Scientific instruments, Inc, Columbia, MD, USA). For fludarabine-G1 and G2, the chromatographic separation was achieved with an ACE Phenyl 3.0 µm packing material, 100 × 4.6 mm (Canadian Life Science, Peterborough, ON, Canada). The mobile phases were solvent A: water with 0.1% formic acid (v/v) and solvent B: methanol, at a flow rate of 0.9 ml/min. Fludarabine- G1 and G2 were eluted using the following programme: 0–2.0 min, isocratic 20% B; 2.0–2.1 min, linear gradient 20–65% B; 2.1–4.0 min, isocratic 65 % B; 4.0–4.1 min, linear gradient 65–90% B; 4.1–5.2 min, isocratic 90 % B; 5.2–5.3 min, linear gradient 90–20 % B; 5.3–8.5 min, isocratic 20% B. For cerdulatinib-G, chlorambucil dechlorinated metabolites G1 and G2, venetoclax-G, and the bendamustine derivative HP2-G, the chromatographic separation was achieved with a Gemini C18 3.0 µm packing material, 100 × 4.6 mm (Phenomenex, Torrance, CA, USA). The mobile phases were solvent A: 1 mM ammonium formate in water and solvent B: 1 mM ammonium formate in methanol at a flow rate of 0.9 ml/min. Cerdulatinib-G and chlorambucil dechlorinated metabolites G1 and G2 were eluted using the following programme: 0–5.0 min, linear gradient 10–90% B; 5.0–5.2 min, isocratic 90% B; 5.2–5.3 min, linear gradient 90–10% B; 5.3–8.3 min, isocratic 10 % B. Venetoclax-G was eluted using the following programme: 0–2.0 min, linear gradient 10–90% B; 2.0-6 min, isocratic 90% B; 6.0–6.1 min, linear gradient 90–10% B; 6.1–9.2 min, isocratic 10% B. The bendamustine metabolite HP2-G, was eluted using the following programme: 0–5.0 min, linear gradient 20–90% B; 5.0–5.2 min, isocratic 90% B; 5.2–5.3 min, linear gradient 90–20% B; 5.3–8.3 min, isocratic 20% B. For idelalisib-G1 and G2, the chromatographic separation was achieved with a Gemini C18 3.0 µm, 100 × 4.6 mm, using solvent A: 2 mM ammonium formate in water and solvent B: 2 mM ammonium formate in methanol at a flow rate of 0.9 ml/min. Idelalisib-G1 and G2 were eluted using the following programme: 0–3.0 min, isocratic 60 % B; 3.0–3.1 min, linear gradient 60–90% B; 3.1–4.0 min, isocratic 90% B; 4.0–4.1 min, linear gradient 90–60% B; 4.1–7.0 min, isocratic 60% B. For ibrutinib-G1 and G2, acalabrutinib-G and duvelisib-G, the chromatographic separation was achieved with an ACE Phenyl 3.0 µm, 100 × 4.6 mm, using a mobile phase of 75%, 75% and 65% methanol, respectively, eluted with 1 mM ammonium formate in water in isocratic mode at a flow rate of 0.9 ml/min. The systems were controlled through Analyst Software, version 1.6.1 from AB Sciex.

### Drug sensitivity assays

Cells were plated at 1 × 10^4^ cells/well (MEC1) or at 5 × 10^4^ cells/well (JVM2) in 96-well U-bottom tissue culture plates (BD Bioscience, Mississauga, ON, Canada). Drugs were added at time of plating at concentrations ranging from 1 nM to 100 µM (7–9 concentrations per drug), depending on the drug and based on cell viability. MTS cell viability assays (Aqueous One assay, Promega, Madison, WI, USA) were conducted 72 h after treatment initiation according to the manufacturer’s instructions. Absorbance was read on a TECAN infinite M1000 plate reader (Tecan Group Ltd., Männedorf, Zurich, Switzerland) at 495 nm. Control cells were treated with corresponding vehicle concentration. To determine half maximal inhibitory concentrations (IC_50_), MTS readout of control samples treated with vehicle only was set to 100% cell viability, and relative cell viability of drug-treated cells was determined by dividing MTS values of the treated samples by the control. IC_50_ were calculated by fitting variable slope non-linear curves to normalised response data from drug treatments using GraphPad Prism v5 (GraphPad Software Inc., La Jolla, CA, USA). Assays were replicated at least three times in triplicates.

Cytotoxicity of drug treatments was determined by labelling cells with AlexaFluor 647-conjugated Annexin V (Life Technologies Inc., CA, USA) and propidium iodide (Sigma). Cells plated in 96-well plates at 2 × 10^4^ cells/well (MEC1) or 3 × 10^4^ cells/well (JVM2) were exposed to varying drug concentrations as above for 72 h. Cells were washed, labelled for 12 min with Annexin V (1:200) and PI (2 µg/ml) in Annexin V binding buffer, then immediately analysed by flow cytometry on a FACSCelesta equipped with a high throughput sampler (BD Bioscience). Annexin V labelling assays were replicated at least twice in duplicates.

### Gene expression analyses

Total RNA was extracted from MEC1, EHEB and JVM2 cells treated with fludarabine, ibrutinib or idelalisib using RNeasy plus mini spin kits (Qiagen, Toronto, ON, Canada). cDNA was generated using SuperScript IV reverse polymerase (Thermo Fisher Scientific, Waltham, MA, USA). *UGT* genes were measured by qPCR analysis of 10 ng of cDNA with *Power* SYBR green master mix (Thermo Fisher Scientific). For measures of other genes including housekeeping genes from normalisation, total RNA was DNase I-treated and purified using the RNeasy MinElute Cleanup kit (Qiagen) per manufacturer’s instructions and as described previously.^22^ RNA sequencing experiments were performed on MEC1, EHEB and JVM2 cells treated with fludarabine (10 µM and 50 µM), ibrutinib (1 µM and 5 µM) and idelalisib (5 µM and 10 µM) for 48 (MEC1), 72 (JVM2) or 96 (EHEB) hours. Sequencing data was quality trimmed using Trimmomatic v0.36 and aligned to GRCh38 using HISAT2 v2.1.^24^ Transcriptome assembly and generation of read-count matrices were performed using Ensembl GRCh38 transcriptome annotation with StringTie v1.3.4.^25^ Differential gene expression analysis was done with the edgeR package for R v3.5.1. Isoform quantification was done using kallisto v0.44.0 with a custom GTF formatted annotation containing alternative UGT transcript information.^26^ Sequencing data are available from the Gene Expression Omnibus (GEO) accession number GSE135030.

Public expression data from 291 CLL patients was downloaded from the International Cancer Genome Consortium (ICGC) with the project code CLLE-ES.^27^ Public microarray data from PBMCs of untreated CLL patients and CD19^+^-sorted B-cells from patients treated with fludarabine-containing regimens were obtained from the Gene Expression Omnibus with the accession numbers GSE13159^28^ and GSE15490,^23^ respectively. In the latter study, responders included complete response or partial response, and non-responders comprised stable disease or progressive disease. Long-read sequencing data was downloaded from the Sequence Read Archive (SRA) using the accession number SRP036136 and aligned with HISAT before visualisation. Identification of possible upstream regulators was carried out using iRegulon^29^ for Cytoscape v3.2.1. ENCODE ChIP-seq data from samples GM12891, GM12878 and GM19099 was analysed for RELA binding within a 10 kb window centred on the TSS of genes co-expressed with *UGT2B17* by bootstrapping. Differential expression analysis for RNA-seq data was done using the edgeR v3.22.5, while microarray data was analysed with limma v3.36.5 and affy v1.58. AnnotationHub v2.12.1, Iranges v2.14.12 and GenomicRanges v1.32.7 were used for analysis of ChIP-seq data. Clustering and functional enrichment analyses were performed using clusterProfiler v3.8.1, coseq v1.4.0, ComplexHeatmap v1.18.1 and DESeq2 v1.20.0 packages for R v3.5.1 (http://www.bioconductor.org).^30–37^

### Luciferase assays

The sequence corresponding to 2.5 kbp of *UGT2B17_n2* promoter (including exon 1c) was PCR amplified from LNCaP cell line genomic DNA using Phusion DNA Polymerase (NEB, Whitby, ON, Canada) as recommended, with the following primers (annealing at 63 °C): forward 5′-CTAGCAGACGCGTGAGATCCTAGTAGGAGGTTTTGGC-3′ and reverse 5′-CTAGCAGCTCGAGCAAGTTCCAGATGTCCAGACTC-3′. The PCR fragment was then digested with MluI and XhoI restriction enzymes and inserted in pGL3 basic vector (Promega) digested with the same enzymes, using the Rapid DNA Ligation Kit (Roche). Construct was verified by Sanger sequencing. MEC1 cells were co-transfected with 9.5 µg pGL3 constructs and 0.5 µg pRL-null basic renilla (Promega) using the Neon Transfection System (Thermo Fisher Scientific). Cells were then harvested, lysed and assessed for luciferase activity using the dual-luciferase reporter assay kit (Promega), as per manufacturer’s instructions. Luciferase activity was calculated as the ratio of firefly luciferase to renilla activity, relative to the pGL3 control. Assays were replicated four times in triplicates.

### Statistical analyses

Results from glucuronidation assays represent a minimum of two independent experiments. All other results represent at least three independent experiments. Statistics were calculated using GraphPad Prism v5 (GraphPad Software Inc., La Jolla, CA, USA) or R v3.5.1. *P*-values were calculated using Student’s *T*-test unless otherwise indicated. Differences in gene expression were considered significant if adjusted *P*-values were inferior to 0.05.

## Results

### UGT2B17 expression in B-cells is associated with reduced sensitivity to anti-leukaemic drugs and is induced by short-term drug treatment in patients

**Fig. 1:**
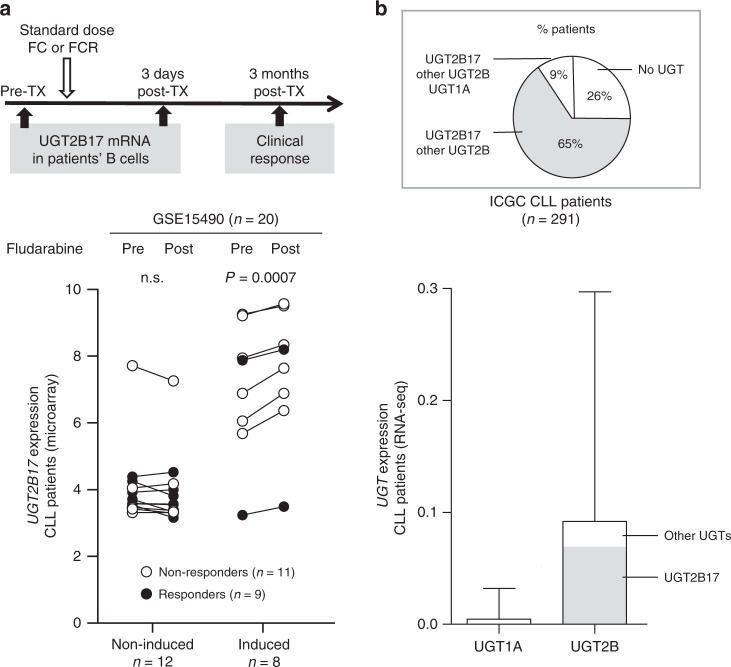
Expression of UGTs in CLL patients and induction of UGT2B17 after treatment initiation in twenty CLL patients receiving fludarabine-containing regimens (GSE 15490).^23^ **a** UGT2B17 is preferentially induced in CLL patients not responding to fludarabine-containing regimen. Expression of UGT2B17 was determined in CD19^+^-sorted cells of CLL patients before and three days after the first cycle of fludarabine treatment. Patients received standard doses of fludarabine and cyclophosphamide (FC) or FC with rituximab (FCR). Clinical response was assessed three months after treatment initiation.^23^ Responders displayed partial or complete remission, non-responders had stable or progressive disease. Expression of *UGT2B17* is significantly enhanced (> 15 %; *P* = 0.0007) three days after treatment in the subset of patients not responding to fludarabine-containing regimen. CLL patients were dichotomised on the basis of *UGT2B17* induction. Prior to initiation of treatment with a fludarabine-containing regimen, average levels of *UGT2B17* were 4.1 and 7.0 log_2_-units (*P* = 0.0006, after normalisation by robust multi-array average) in patients exhibiting no induction and induction of *UGT2B17* expression, respectively. Clinical characteristics of CLL patients are provided in Supplementary Table S6. **b** *UGT2B17* predominates in leukaemic cells. Relative expression levels of UGT isoforms in a cohort of 291 CLL patients from the International Cancer Genomic Consortium (ICGC) demonstrate that UGT2B17 is the main UGT expressed in leukaemic cells. The proportion of patients expressing UGT2B17 and other UGTs is illustrated. Other UGTs include nine UGT1A (1A1, 1A3, 1A4, 1A5, 1A6, 1A7, 1A8, 1A9 and 1A10) and six UGT2B (2B4, 2B7, 2B10, 2B11, 2B15 and 2B28).

In 20 CLL patients exposed to fludarabine-based regimens,^23^ microarray data analysis of RNA from CD19^+^-sorted B-cells (GSE15490) showed that *UGT2B17* was induced shortly after treatment initiation in 6 of 11 non-responders whereas it was induced in only 2 responders out of 9 (*P* = 0.0007) (Fig. 1a). Patients showing an induced expression of *UGT2B17* after treatment also displayed a 7.5-fold higher basal expression of *UGT2B17* relative to those showing no induction (*P* = 0.0006), with all cases showing low expression of all other UGT isoforms. This was sustained by the analysis of RNA sequencing data from 291 CLL cases indicating that UGT2B17 predominates in leukaemic cells (Fig. 1b). Cells of MEC1 and JVM2 models overexpressing UGT2B17 were more resistant than cells with low expression, with half maximal inhibitory concentration (IC_50_) values higher by 1.5 to 4.3-fold (*P* < 0.013) for fludarabine and ibrutinib (MEC1 and JVM2) as well as idelalisib, chlorambucil and venetoclax (MEC1) established by MTS assays (Table 1). Similar results were obtained using AnnexinV/PI labelling (data not shown). At clinically relevant concentrations, fludarabine, ibrutinib and idelalisib displayed no cytotoxicity in either cell lines, determined by Annexin V staining (Supplementary Fig. S1).

**Table 1:** Drug sensitivity of lymphoid cell models overexpressing UGT2B17 compared to control cells.

	Fludarabine	Ibrutinib	Idelalisib	Venetoclax	Chlorambucil	Bendamustine
*MEC1 cells*						
CTRL IC_50_ (µM)	29.27	0.99	1.73	2.22	7.99	60.09
2B17 IC_50_ (µM)	58.82	4.13	7.40	4.16	12.35	76.05
*P*	<**0.001**	**<0.001**	**0.014**	**0.025**	**<0.001**	0.094
Fold over control	2.01	4.17	4.28	1.87	1.55	1.27
*JVM2 cells*						
CTRL IC_50_ (µM)	9.08	3.64	1.79	4.06	10.38	54.24
2B17 IC_50_ (µM)	15.05	8.95	2.95	2.94	8.64	62.78
*P*	<**0.001**	**0.013**	0.588	0.100	0.278	0.233
Fold over control	1.66	2.46	1.65	0.72	0.83	1.16

Half-maximal inhibitory concentrations (IC_50_) are shown for cells overexpressing UGT2B17 (2B17) or controls (CTRL). Cell viability was tested by a colorimetric (MTS) assays with at least seven drug concentrations, ranging from 1 nM to 100 µM (distinct range for each drug) to establish the IC_50_. *P* values below 0.05 were considered statistically significant (in bold). Assays were replicated at least three times in triplicates. Similar results were obtained using AnnexinV/PI labelling (data not shown).

### UGT2B17 expression in B-cell models is induced by short-term drug treatment

**Fig. 2:**
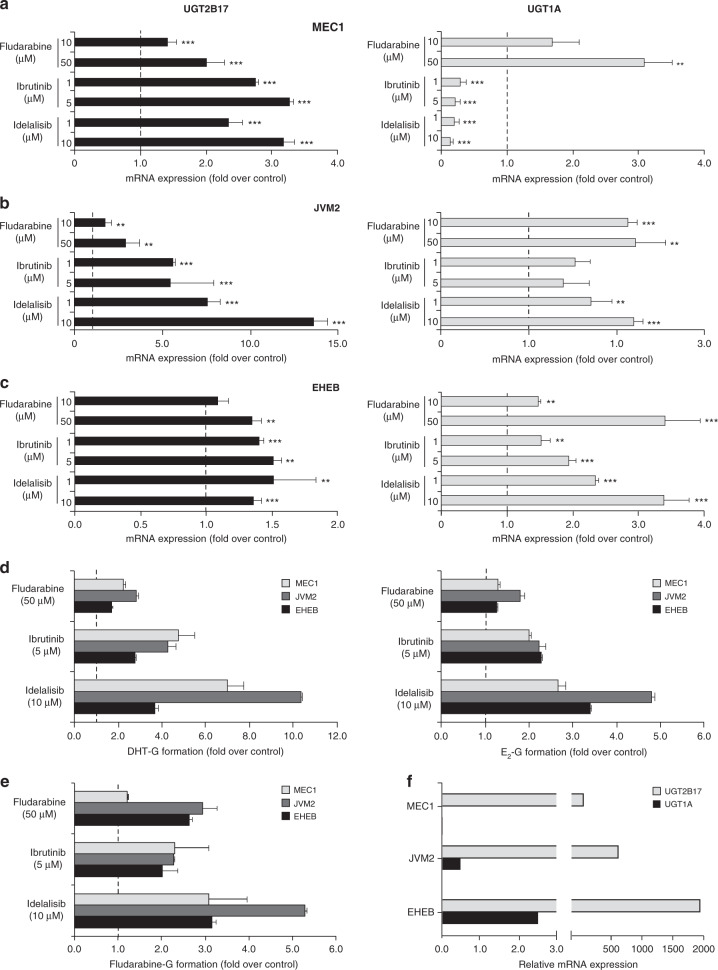
Drug treatments induced UGT2B17 expression and activity in CLL cell models. Quantification of *UGT2B17* (left panels) and *UGT1A* (right panels) mRNAs by RT-qPCR in MEC1 (**a**), JVM2 (**b**) and EHEB (**c**) cells subjected to fludarabine, ibrutinib or idelalisib treatment at specified concentrations. **d** Drug treatments induced UGT2B17 (left) and UGT1A (right) glucuronidation activity for their probe substrates dihydrotestosterone (DHT) and oestradiol (E_2_), respectively. **e** Fludarabine glucuronidation was enhanced following treatment with fludarabine, ibrutinib or idelalisib. **f** Relative expression levels (×10^3^) of *UGT1A* and *UGT2B17* in leukaemic cell models. G Glucuronide. Student’s *T*-test; *P < 0.05, **P < 0.01, ***P < 0.001.

As observed in CLL patients (Fig. 1a), fludarabine significantly induced *UGT2B17* expression in MEC1, JVM2, and EHEB cellular models (Fig. 2a–c). UGT2B17 was also induced by ibrutinib and idelalisib (by 1.2 to 13.6-fold; *P* < 0.01) and was associated with elevated enzyme activity for DHT glucuronidation, a substrate for the UGT2B17 enzyme (1.9 to 10.5-fold; *P* < 0.01) (Fig. 2d). The low expression of UGT1A was also perturbed by each drug treatment (Fig. 2a–c). Consistent with these changes, glucuronidation activity was induced as demonstrated by increased levels of conjugate of the substrate oestradiol (E_2_) (Fig. 2d). In the same line, fludarabine, ibrutinib and idelalisib increased fludarabine glucuronidation (Fig. 2e). As observed in CLL patients (Fig. 1b) comparative analysis of relative UGT expression levels further indicated a predominance of UGT2B17 (Fig. 2f). A more detailed assessment of the isoforms expressed identified UGT1A6 as the most abundant UGT1A isoform (Supplementary Fig. S2).

### Anti-leukaemic drugs are conjugated to GlcA in CLL-treated patients

**Fig. 3:**
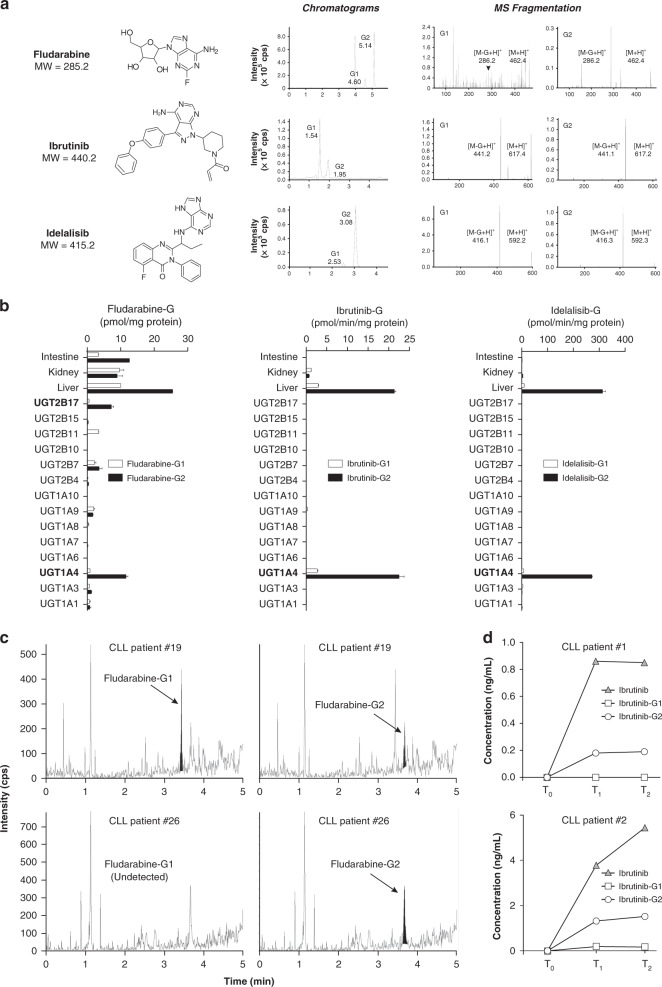
Inactivation of anti-leukaemic drugs by glucuronidation in human metabolic tissues, B-cell models, and detected in CLL patients. **a** Fludarabine, ibrutinib or idelalisib glucuronidation by human liver microsomes generated two glucuronidated metabolites G1 and G2 for each drug separated by liquid chromatography and named according to their order of elution. MS fragmentation patterns confirmed the glucuronidated nature of metabolites with the loss of the glucuronic acid (GlcA) moiety corresponding to a m/z shift of 176 Da. Masses of the protonated drug-glucuronide [M + H]^+^ and parent drug [M-G + H]^+^ are shown and were in accordance with those of the parent drugs alone and of GlcA. Their identity was confirmed upon β-glucuronidase hydrolysis (not shown). **b** Identification of UGT2B17 and UGT1A4 enzymes primarily targeting anti-leukaemic drugs for glucuronidation using quantitative MS methods. The screening included human liver, intestine and kidney that are enriched in UGTs and individual recombinant UGT1A (*n* = 8) and UGT2B (*n* = 6) enzymes. **c** Detection of fludarabine-G in plasma samples of two CLL patients collected in the first week after fludarabine treatment. Peaks corresponding to fludarabine-G are highlighted. **d** Concentrations of ibrutinib-G and the parent drug measured by quantitative MS in two CLL patients undergoing ibrutinib treatment collected at baseline (T_0_), 3 weeks to 2 months (T_1_) and between 4 and 9 months (T_2_) after treatment initiation. Patients’ characteristics are displayed in Table S4.

Given the higher and induced expression of UGT2B17 in patients not responding to fludarabine-containing treatment regimen, we explored whether UGT2B17 may be involved in the glucuronidation of the drug itself. We also examined the conjugation of additional agents relevant to CLL. Our observations in CLL patients treated with fludarabine raised the possibility that UGT2B17 may be involved in the glucuronidation of the drug itself, and potentially also of B-cell receptor inhibitors ibrutinib and idelalisib, which contain functional hydroxyl and amino groups susceptible for conjugation into inactive glucuronides (G). Glucuronidation assays using pooled human liver microsomes enriched in UGT enzymes led to the formation of polar G derivatives. This was confirmed by their fragmentation patterns assessed by mass spectrometry (MS), namely a loss of the GlcA moiety corresponding to a m/z shift of 176 Da (Fig. 3a). Fludarabine was conjugated into two glucuronides G1 and G2, named according to their chromatographic resolution (Fig. 3a). Additional confirmation was achieved by the disappearance of G1 and G2 upon β-glucuronidase hydrolysis (Supplementary Figure S3). Similarly, ibrutinib and idelalisib each led to two glucuronidated products (Fig. 3a).

**Fig. 4:**
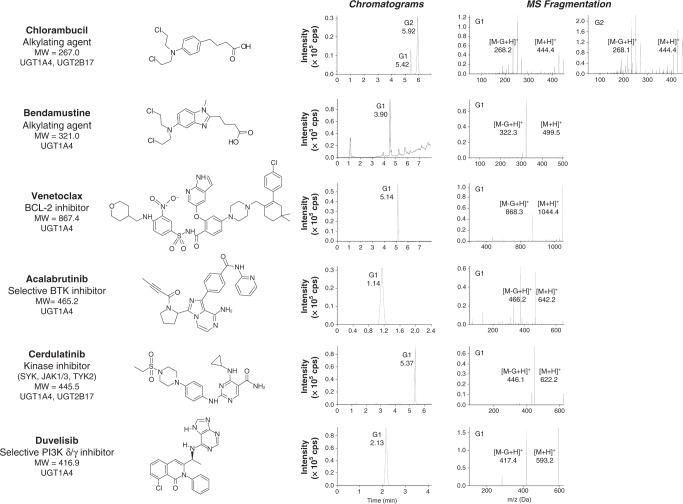
Anti-leukaemic agents used in the treatment of CLL are substrates for UGT-dependent glucuronidation. MS analysis indicated the formation of one or two glucuronidated products named according to their order of elution G1 and G2. Glucuronidation assays using pooled human liver microsomes enriched in UGT enzymes led to the formation of polar G derivatives confirmed by their fragmentation patterns assessed by mass spectrometry (MS), namely a loss of the GlcA moiety corresponding to a m/z shift of 176 Da. Masses of the protonated drug-glucuronide [M + H]^+^ and parent drug [M-G + H]^+^ are shown and were in accordance with the masses of the parent drug and of GlcA (176 Da). The metabolite of bendamustine HP2 was glucuronidated. The HP2 metabolite has hydroxyl groups in place of chlorine atoms in bendamustine. Chlorambucil undergoes a similar process, generating a dechlorinated metabolite subsequently conjugated. Venetoclax is an orally available, selective, small molecule inhibitor of BCL2 approved by the US Food and Drug Administration for the treatment of patients with CLL. Acalabrutinib is an orally available, irreversible Bruton’s tyrosine kinase (BTK) inhibitor in development designed to be more selective than ibrutinib.^10^ Cerdulatinib (PRT062070) is an investigational oral, dual spleen tyrosine kinase (Syk), janus kinase (JAK1/3) and tyrosine kinase 2 (TYK2) inhibitor for the treatment of haematological malignancies and approved for the treatment of peripheral T-cell lymphoma.^9^ Duvelisib is an oral, dual small molecule inhibitor of phosphatidylinositol 3-kinase (PI3K) δ and γ, approved for the treatment of relapsed or refractory CLL.^7^ A second set of experiments identified the UGT enzyme(s) involved, revealing a predominant role for UGT2B17 and UGT1A4.

A second set of experiments identified the UGT enzyme(s) involved, based on UGT expressed in metabolic liver, kidney and intestine tissues, and individual recombinant UGT1A (*n* = 8) and UGT2B (*n* = 6) enzymes, using quantitative MS methods (Fig. 3b). UGT2B17 and UGT1A4 were the main conjugating enzymes for fludarabine glucuronidation (Fig. 3b), with a preferred formation of G2 over G1. This observation mirrored activity in livers, which express these UGTs. The confirmation of fludarabine-G formation in CLL patients was established in plasma of two cases collected in the first week after fludarabine treatment (Fig. 3c). Both fludarabine-G1 and G2 were measured in the first patient (16.4 and 11.7 pg/mL), whereas G2 (24.1 pg/mL) was detected in the second patient. For ibrutinib and idelalisib, G1 and G2 were formed only in the presence of UGT1A4 and livers, with similar kinetic parameters (Fig. 3b, Supplementary Table S1), supporting the implication of this sole enzyme. Their formation was further abolished by nearly 90% in the presence of a specific UGT1A4 inhibitor, hecogenin (Supplementary Table S1). The formation of ibrutinib-G was then established in serial serum samples from 15 CLL patients undergoing ibrutinib treatment collected at baseline (T_0_), 3 weeks to 2 months (T_1_), and between 4 and 9 months (T_2_) after treatment initiation (Fig. 3d, Supplementary Tables S2 and S3). A predominance of G2 over G1 (ratio of 6.5) was noted, similar to what was observed in livers and UGT1A4. The formation of ibrutinib-G represented on average 24 % of the parent drug and was strongly correlated to levels of ibrutinib (*R*^2^ = 0.917, *P* < 0.001) but with a considerable patient-to-patient variability (CV = 190%). Lastly, we explored whether glucuronidation may be involved in the conjugation of additional anti-leukaemics and the potential involvement of UGT2B17. MS analysis confirmed the formation of at least one G product by livers following incubations with bendamustine, chlorambucil and targeted agents venetoclax, acalibrutinib, cerdulatinib and duvelisib (Fig. 4). The UGT2B17 and UGT1A4 enzymes were predominantly involved in their inactivation by glucuronidation (Fig. 4).

### Transcriptional changes associated with high UGT2B17 in CLL patients and cell models

**Fig. 5:**
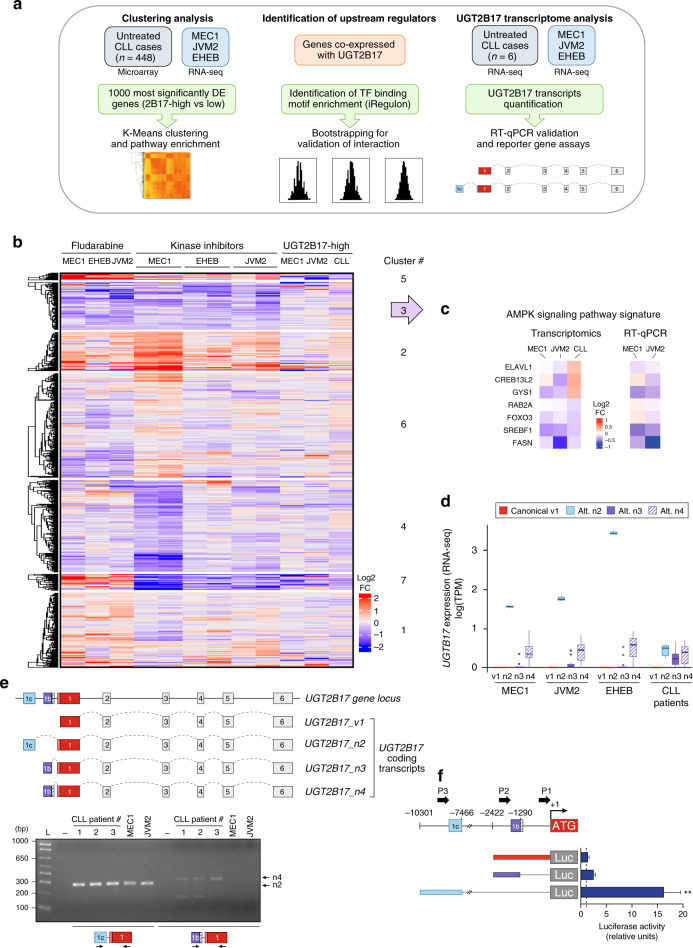
Ascertaining pathways affected by high UGT2B17 expression in B cells and upstream regulators. **a** Schematic overview of the analysis pipeline. K-means clustering, enrichment and co-expression analyses served to identify pathways associated with high *UGT2B17* expression and upstream transcriptional regulators. Detailed analysis of *UGT2B17* transcriptome by RNA-seq revealed expression of non-canonical transcripts and alternate regulation. **b** A transcriptional signature associated with elevated *UGT2B17* expression in PBMCs from 448 untreated CLL patients (GSE13159)^28^ enabled clustering and identification of pathways altered by elevated UGT2B17 expression. The top 1000 most statistically significant genes were selected as features for clustering RNA-seq samples with log2 fold-change values using the K-means method. Cluster 3 contained genes which were globally down-regulated across all experimental conditions and was enriched with genes belonging to the AMPK signalling pathway. **c** RT-qPCR validation of down-regulated AMPK pathway-related genes in cell models. **d** UGT2B17 is predominantly expressed from an alternative *UGT2B17_n2* transcript in lymphoid cell models and in CLL patients (*n* = 6; GSE99724) evaluated by RNA sequencing. **e** Schematic overview of the *UGT2B17* gene and main coding transcripts in leukaemic cells. Only exons included in main leukaemic *UGT2B17* transcripts are shown for sake of clarity. The alternative *UGT2B17_n2-n4* transcripts include the supplementary exon 1c or 1b previously reported, which extend the 5′ untranslated sequence relative to the canonical *UGT2B17_v1* transcript.^26^ *UGT2B17_n2* and *n4* transcripts were validated by RT-PCR in cells of three CLL patients, and in MEC1 and JVM2. These transcripts encode a functional UGT2B17 enzyme. **f** Luciferase reporter gene expression assays were performed in MEC1 cells. Cells were transfected with pGL3 vectors containing either the canonical promoter of UGT2B17_v1 (P1), or the alternative promoters P2 or P3 upstream of each novel exons 1b or 1c, respectively. Experiments were conducted four times in triplicates.

To gain insights into the cellular pathways associated with high and inducible UGT2B17 expression, we initially established a transcriptional signature associated with elevated *UGT2B17* expression in 448 untreated CLL samples (Fig. 5a). This signature was then examined in cell models expressing high levels of UGT2B17 as well as in drug-treated cells in which UGT2B17 expression was induced. First, clustering revealed transcriptomic changes associated with elevated *UGT2B17* expression in untreated CLL patients that resembled those found in overexpression models (Fig. 5b). K-means clustering of gene expression data designated two clusters with globally up-regulated (cluster 2) or down-regulated (cluster 3) gene expression across all samples, suggesting that these clusters encompass changes connected to UGT2B17 levels rather than those produced by drugs. Cluster 2 contained the *UGT2B17* gene whereas several genes of the AMP-activated protein kinase (AMPK) signalling pathway were significantly enriched in cluster 3 (Supplementary Table S4), and further validated by quantitative PCR (Fig. 5b, c).

A second series of analysis focused on drug-related signatures. We observed that kinase inhibitor-treated cells clustered together whereas fludarabine-treated cells had a distinct expression profile, likely owing to different mechanisms of action (Fig. 5b). An upstream analysis for causal interpretation of the expression changes associated with UGT2B17 exposed an enrichment of nuclear factor kappa B (NF-κB) binding targets (Supplementary Table S5). The analysis of NF-κB ChIP-seq data (GM12891, GM12878 and GM19099) derived from tumour necrosis factor α (TNF-α)-treated B-cells further confirmed NF-κB as a key regulatory ‘hub point’.

An analysis of the *UGT2B17* transcriptomes of CLL patients and leukaemic cell models revealed that the enzyme is largely expressed from alternative transcripts rather than the canonical *v1* transcript (Fig. 5d, e). Although these alternative transcripts, named *UGT2B17_n2*, *n3* and *n4*, encode the UGT2B17 enzyme, they are comprised of additional alternative exons that extend the 5’ untranslated region (Fig. 5e). Their expression was also detected by RT-PCR amplification of full-length transcripts from CLL patients and Sanger sequencing of amplicons. Long-read sequencing data using PacBio SMRT technology also confirmed the expression of alternative *UGT2B17_n2* in the GM12891 lymphoid cell line. The functionality of the regulatory sequences upstream of the novel exon 1c (P3) and exon 1b (P2) were evidenced in luciferase assays (Fig. 5f). Compared to the low expression derived from the canonical *UGT2B17* promoter P1 (1.3-fold), P2 and P3 enhanced luciferase gene expression by 2.5- and 16.3-fold, respectively, in MEC1 cells (Fig. 5f).

## Discussion

Understanding mechanisms that contribute to intrinsic and acquired resistance to therapy is key to finding useful predictive markers and innovative strategies to prevent or overcome treatment resistance in CLL. Previous reports identified UGT2B17 as a prognostic marker and a potential therapeutic target.^17,20,21,38^ A potential impact of UGT2B17 expression on clinical outcome in treated patients emerges with observations of poor response in fludarabine-treated patients expressing high UGT2B17 levels whereas drug treatment further induces the UGT2B17-associated metabolic capacity of lymphoid cells according to our findings. Our observations further support a significant impact of the UGT metabolic pathway on the inactivation of most anti-cancer agents used in CLL, including commonly used treatments fludarabine, ibrutinib, bendamustine, chlorambucil and emerging targeted therapies idelalisib, venetoclax, acalabrutinib, cerdulatinib and duvelisib. This resulted in reduced sensitivity of cells to several anti-leukaemics associated with high UGT2B17 expression. Our findings indicate that this may be caused, at least in part, by direct glucuronidation of the drug by the UGT2B17 enzyme namely for fludarabine but would also involve other mechanisms for ibrutinib and idelalisib not inactivated by UGT2B17.

By catalysing the transfer of GlcA from the co-substrate UDP-GlcA, UGT2B17 inactivates and detoxifies its substrates. This is supported by the detection of fludarabine-glucuronides in circulation of CLL patients that recently initiated fludarabine-based treatment. The glucuronidation pathway inactivates other nucleotide analogues such as ribavirin and cytarabine through a glioma-associated oncogene homologue 1 (GLI1)-dependent mechanism involving the regulation of UGT1A protein stability in AML.^18,19^ This differs from our observations in untreated CLL patients, in which UGT2B17 expression predominates, and where high UGT2B17 expression was associated with shorter treatment-free and overall survival and more patients requiring treatment.^17,20,21^ This shows that for a significant proportion of high-risk CLL patients, UGT2B17 is expressed in cancer cells prior to treatment initiation with the potential to affect primary response to first line treatment such as fludarabine and ibrutinib. Once fludarabine treatment is initiated, an induction of UGT2B17 expression was observed in B-cells of CLL patients not responding to fludarabine as well as in lymphoid cell models.^17,23^

Treatment with targeted agents ibrutinib and idelalisib also resulted in a marked transcriptional up-regulation of *UGT2B17*, and high UGT2B17 expression was associated with reduced sensitivity to these drugs in B-cell models. While an influence of other drug-related mechanisms, including transporters and drug metabolising enzymes is possible, CYP3A4, which is another key metabolising enzyme for these drugs,^39^ was not perturbed in patients treated with fludarabine (not shown). It raises the possibility that therapeutic pressure induces UGT expression in B-cells. This rapid adaptation of neoplastic cells in the presence of a cytotoxic stressor and targeted therapies support that the UGT metabolic pathway is highly relevant in leukaemia, has the potential to affect drug response locally in malignant cells, and may be useful in predicting response to several CLL therapies. We also demonstrated that the glucuronidation pathway is involved in the conjugation of a number of additional anti-leukaemics (chlorambucil, bendamustine, venetoclax, acalabrutinib, cerdulatinib and duvelisib) and that UGT2B17 plays a role in the inactivation of chlorambucil and cerdulatinib. These two anti-leukaemic agents target different cellular pathways than fludarabine and have distinct modes of action. High UGT2B17 expression may thus lead to lower response to these drugs in CLL patients but this remains to be demonstrated.

Crucial parts of the machinery governing *UGT2B17* transcription remain poorly understood and especially in lymphoid cells. We provide the first evidence that *UGT2B17* expression in B-cells is driven by a non-canonical *UGT2B17* promoter and the use of an alternative noncoding exon 1c coupled to the common protein-coding region leading to the canonical UGT2B17 enzyme. According to our analysis, the gene expression signature associated with high UGT2B17 expression in CLL patients and cell models comprises a number of genes targeted by NF-κB. This promoter may thus be targeted by NF-κB that plays a central role in CLL. The NF-κB pathway is associated with poor prognosis and response to drug treatment, and represents an emerging drug target in CLL.^17,40–44^ The mechanism underlying high UGT2B17 expression in B-cells remains to be fully explored.

Our data also point to other mechanisms of altered drug sensitivity associated with high UGT2B17 expression. The inactivation of ibrutinib and idelalisib is largely dependent on UGT1A4 as the conjugation of other anti-leukaemics tested herein (chlorambucil, bendamustine, venetoclax, acalabrutinib, cerdulatinib and duvelisib). UGT1A4 is far less abundant than UGT2B17 in leukaemic B cells, and undetected at the mRNA level in most untreated CLL patients. Given that ibrutinib is mainly administered as an oral agent and subjected to first-pass hepatic metabolism, this drug is likely conjugated primarily in the liver expressing high levels of UGT1A4. In one of the lymphoid cell models, we showed drug-mediated induction of UGT1A4, but this finding remains to be demonstrated in CLL patients. Induction at the protein level also requires examination, given the recent report in drug-resistant AML cells suggesting reduced mRNA expression but an enhanced UGT1A protein expression mediated by increased protein stability upon drug treatment.^18^

Consistent with its regulatory function of the levels of endogenous molecules, high UGT2B17 may deplete intracellular metabolites leading to aberrant cell signalling and dysregulated cell functions,^22^ which could favour progression in untreated CLL cases and subsequent drug resistance in treated patients. In our transcriptomic analysis, AMPK signalling was negatively associated with high UGT2B17 expression in conditions of both induced and high basal UGT2B17 expression in cell models and CLL patients, potentially linking B-cell metabolism to the glucuronidation pathway and subsequent adverse clinical outcomes. AMPK is a major regulator balancing energy supply and ultimately protects cells from harmful stresses by the coordination of multiple metabolic pathways.^45^ The activation of the AMPK pathway has been shown to affect growth and apoptosis in CLL.^46^ As a stress-response molecule mediating drug resistance through different mechanisms, AMPK is further involved in the metabolism reprogramming and induction of autophagy, also regulating the self-renewal ability of cancer stem cells.^47^ More recently, ibrutinib resistance was associated with a metabolic rewiring in CLL.^48^ Likewise, UGT proteins were shown to be part of complex protein networks. Their functional interaction with other metabolic proteins induced broad changes in cell metabolism and may contribute to tumorigenesis and drug response.^49,50^

The findings of this study have to be seen in light of some limitations, including the fact that drug cytotoxicity and treatment outcome in relation to UGT2B17 expression was investigated in cell models and a limited number of fludarabine-treated patients. However, IC_50_ values of tumour cells, namely for ibrutinib, were in the same range as those reported previously in B lymphoblastoid leukaemic cell lines and in primary cells from patients,^51–53^ supporting the relevance of our initial observations. The impact of UGT2B17 expression on response to drug in vivo and ex vivo, as well as the influence of the microenvironment on UGT2B17-related responses, remains to be examined in more details.

In summary, we unveiled a biochemical underpinning of reduced drug sensitivity related to the UGT2B17 metabolic pathway and drug inactivation. The evidence provided should prove useful for understanding and potentially overcoming drug refractoriness. The impact of glucuronidation in the inactivation of a number of anti-leukaemic drugs is underestimated since we established that most agents are subjected to this metabolic process, including ibrutinib, idelalisib, venetoclax and duvelisib as well as other small molecules under development such as acalibrutinib and cerdulatinib. This may well apply to a number of other cancer therapeutics given the recent report that GLI1-inducible glucuronidation imparts resistance to a broad spectrum of compounds including FDA-approved drugs such as methotrexate.^19^ Our observations warrant additional studies to appreciate the prevalence and the clinical implications of high UGT2B17 expression on outcomes of leukaemia patients.

## Supplementary information

Supplementary information

## Data Availability

The datasets generated during the current study are available in the Gene Expression Omnibus repository with the accession number GSE135030.
