# Supplementary material for: UGT2B17 modifies drug response in chronic lymphocytic leukaemia
Source: Br J Cancer. 2020 May 18;123(2):240–51. doi: 10.1038/s41416-020-0887-6 (PMC7374097; doi:10.1038/s41416-020-0887-6)

Supplementary information for:

UGT2B17 Modifies Drug Response in Chronic Lymphocytic Leukaemia

Eric P. Allain[1], Michèle Rouleau[1], Katrina Vanura[2], Sophie Tremblay[1], Joanie Vaillancourt[1], Vincent Bat[1], Patrick Caron[1], Lyne Villeneuve[1], Adrien Labriet[1], Véronique Turcotte[1], Trang Le[2], Medhat Shehata[2], Susanne Schnabl[2], Dita Demirtas[2], Rainer Hubmann[2], Charles Joly-Beauparlant[3], Arnaud Droit[3], Ulrich Jäger[2], Philipp B. Staber[2], Eric Lévesque[4], Chantal Guillemette[1,5]

Table S1 Kinetic estimates for conjugation of fludarabine, ibrutinib and idelalisib by human liver and UGT enzymes and inhibitory constants (K_i) for drug glucuronidation.

**Protein preparation**	**Affinity K_m (µM)**			**Velocity V_{max} (pmol/min/mg protein)**			**UGT1A4 inhibitory constant K_i (µM)**		
				Fludarabine					
Liver	878	±	15	0.12	±	0.03	-		
UGT2B17	949	±	4	0.01	±	0.00	-		
UGT1A4	1020	±	7	0.05	±	0.00	-		
				Ibrutinib					
Liver	39	±	6	90	±	14	5.8	±	0.2
UGT1A4	58	±	13	68	±	2	2.8	±	0.4
				Idelalisib					
Liver	54	±	1	530	±	44	0.9	±	0.1
UGT1A4	45	±	3	348	±	1	0.6	±	0.0

Observed V_{max} values correspond to the velocity measured at the highest concentrations of substrate. Affinities (K_m) were estimated by calculating ½ of the observed V_{max}. Hecogenin was used as a specific UGT1A4 inhibitor. Values are only shown for the predominant drug-G. - : not determined.

Table S2 Characteristics of CLL patients under ibrutinib treatment.

Characteristics	**N = 15 (%)**
Sex	
Male	8 (53)
Female	7 (47)
Binet stage B/C	6 (40)
CD38 High	4 (27)
Del(13q)	8 (53)
Tris 12	5 (33)
Del(11q)	4 (27)
Del(17p)	4 (27)
UM IGHV	10 (67)

Table S3 MS-based quantification of ibrutinib and ibrutinib-G in plasma samples of 15 CLL patients undergoing ibrutinib therapy.

Patients (n = 15)	Ibrutinib	Ibrutinib-G1	Ibrutinib-G2	Total ibrutinib-G	G / Ibrutinib
Mean (ng/mL)	12.72	0.54	3.51	4.05	24%
Median (ng/mL)	20.73	1.02	6.67	7.69	12%
SD (ng/mL)	3.68	0.20	0.99	1.24	24%
CV (ng/mL)	163%	190%	190%	190%	52%

Data are derived from blood samples collected after at least 4 months of therapy (at T_2) and data were highly similar for T_1. CV = coefficient of variation; SD standard deviation; G= glucuronide.

Table S4: Major pathways associated with UGT2B17 expression defined by KEGG classifications based on their expression pattern by K-mean clustering and their significance determined using a hypergeometric test (False discovery test < 0.05 are indicated in bold).

Cluster #	Description of expression changes	Major enriched pathways
1	Little to no change across all samples.	**Valine, leucine and isoleucine degradation**<br>**Propanoate metabolism**<br>Pantothenate and CoA biosynthesis<br>Glycosylphosphatidylinositol (GPI)-anchor biosynthesis<br>Fructose and mannose metabolism
2	Up-regulated in all samples.	Steroid hormone biosynthesis<br>Metabolism of xenobiotics by cytochrome P450<br>Ascorbate and aldarate metabolism<br>Drug metabolism - other enzymes<br>Chemical carcinogenesis
3	Down-regulated in all samples.	**AMPK signaling pathway**<br>Mitophagy - animal<br>Renal cell carcinoma<br>Ras signaling pathway<br>Insulin resistance
4	Down-regulated in the MEC1 cell model treated with kinase inhibitors.	Phosphatidylinositol signaling system<br>Inositol phosphate metabolism<br>Notch signaling pathway<br>Thyroid hormone signaling pathway<br>Shigellosis
5	Up-regulated in conditions of all drug treatments (cell models and patients).	**Hematopoietic cell lineage**<br>Legionellosis<br>Inflammatory bowel disease (IBD)<br>Leishmaniasis<br>Pertussis
6	Down-regulated in fludarabine-treated conditions (cell models and patients)	Purine metabolism<br>Ribosome<br>Base excision repair<br>Primary immunodeficiency<br>Protein processing in endoplasmic reticulum
7	Up-regulated in fludarabine-treated cells, down-regulated in all other conditions.	Amoebiasis<br>JAK-STAT signaling pathway<br>PI3K-Akt signaling pathway<br>Transcriptional misregulation in cancer<br>Focal adhesion

Table S5. NF-κB gene targets co-expressed with UGT2B17 during drug treatment.

Genes regulated by NF-κB according to recent studies that are co-expressed with UGT2B17 in CLL patients treated with a fludarabine-containing regimen and in leukaemic cell models MEC1 and JVM2 after treatment with fludarabine, ibrutinib or idelalisib

	Cell lines			CLL patients		
**Study**	**Gilmore**	**Li**	**Zhao**	**Gilmore**	**Li**	**Zhao**
**NF-κB-regulated genes**	CXCL3	ALOX5	OR7E47P	CCL3	ABR	AHNAK
	TFF3	ATOX1	EPB41L4A	CD38	AHNAK	ZBTB32
	TNFRSF1B	BCL2L11	SACS	NOD2	AICDA	LDLRAD4
	HAMP	CD74	RIN3	GCLC	APBB2	IL6R
	ALOX5	CDK6	OSBPL10	ADORA2A	ATOX1	RXRA
	NQO1	CXCL3	TCF7	BNIP3	AZIN1	GALNT2
	GRM2	FCGBP		KLF10	BCAT1	RHOBTB2
	BCL2L11	GRM2		MYB	BNIP3	TRIB1
	CDK6	HAMP		AICDA	BTK	ABCG1
	NUAK2	IKBKE		BTK	CCND2	SH3PXD2A
	SERPINB1	INO80C		CTSB	CD38	GABPB1
		NEO1		DPYD	CD59	CSTB
		NQO1		MYLK	CDK12	ABR
		PLEKHG2		CCND2	CEACAM1	ELL3
		PTGER4		LGALS3	CPD	HSP90B1
		RAPH1		SLC16A1	CTSB	CTNNA1
		SERPINB1		PTEN	CTSS	WARS
		TFF3		TICAM1	CYFIP1	
		TNFRSF10B			DPYD	
		TNFRSF1B			DSTN	
		TPMT			FBXO46	
					FBXW11	
					GABPB1	
					GCH1	
					GCLC	
					IGSF3	
					IL15RA	
					KLF10	
					KRT18	
					LGALS3	
					MAPK14	
					MTMR2	
					MYB	
					MYLK	
					NOD2	
					NUMB	
					PANX1	
					PFKP	
					PIGR	

PTEN
PTK6
PTPN2
PTTG1IP
RAPGEF3
RCAN1
RUSC1
SAR1B
SERPINB8
SERPINH1
SH3BP4
SLC16A1
SLC29A1
SRGN
ST7
STAT5B
TICAM1
TNFAIP1
TP53I3
TSG101

References:

Gilmore T, The Gilmore Lab Gene Resources, NF-κB Target Genes,
https://www.bu.edu/nf-kb/gene-resources/target-genes/
Accessed June 2019

Li X, Zhao Y, Tian B, Jamaluddin M, Mitra A, Yang J, Rowicka M, Brasier AR, Kudlicki A.
Modulation of gene expression regulated by the transcription factor NF-κB/RelA.
J Biol Chem 289:11927-11944 (2014).

Zhao M, Joy J, Zhou W, De S, Wood WH 3rd, Becker KG, Ji H, Sen R.
Transcriptional outcomes and kinetic patterning of gene expression in response to NF-κB activation.
PLoS Biol 16:e2006347 (2016).

Table S6: Characteristics of CLL patients treated with fludarabine-containing regimen

Patient ID	Age	Sex	IgHV mut.[1]	Cytogenetic[2]	Treatment[3]	Response[4]	UGT2B17 Induction[5]
CLL1	49	M	UM	Normal	FC	NR (PD)	I
CLL2	63	M	M	13q-(78%)	FC	NR (SD)	I
CLL3	65	M	M	13q-(61%),17p-(73%)	FC	R (PR)	NI
CLL4	65	M	M	13q-(87%)	FC	R (PR)	NI
CLL5	77	M	M	13q-(35%)	FC	R (PR)	NI
CLL6	61	F	UM	13q-(94%)	FC	R (PR)	I
CLL7	75	F	UM	12+(26%)	FC	NR (SD)	NI
CLL8	69	M	UM	Normal	FC	NR (SD)	I
CLL9	74	M	UM	11q-(10%), 12+(47%)	FC	NR (SD)	I
CLL10	68	M	M	13q-(88%)	FC	NR (SD)	NI
CLL11	63	M	M	13q-(48%), 12+(8%)	FCR	R (PR)	NI
CLL12	57	M	NA	13q-(53%), 12+(15%)	FCR	R (PR)	I
CLL13	60	M	UM	13q-(72%), 11q-(8%)	FCR	NR (PD)	NI
CLL14	54	M	UM	11q-	FCR	R (PR)	NI
CLL15	45	M	UM	Normal	FCR	R (PR)	NI
CLL16	63	M	UM	13q-(64%), 11q-(40%)	FCR	NR (SD)	I
CLL17	50	M	UM	11q-(88%)	FCR	NR (SD)	I
CLL18	54	M	UM	NA	FCR	NR (SD)	NI
CLL19	59	F	NA	12+(21%)	FCR	R (PR)	NI
CLL20	56	M	M	13q-(79%),17p-(59%), 11q-(23%)	FCR	NR (SD)	NI

[1] UM: unmutated; M: mutated

[2]13q-, 13q14 deletion; 17p-, 17p13/TP53 deletion; 11q-, 11q22/ATM deletion; 12+, trisomy 12; NA, not available.

[3]FC: fludarabine+cholorambucil; FCR: fludarabine+chlorambucil+ rituximab

[4]Response was determined according to the consensus guidelines of the international workshop on chronic lymphocytic leukemia (iwCLL);

Responders (R): PR, partial remission; Non-responders (NR): PD, progressive disease; SD, stable disease

[5]Gene was considered induced if expression was >15% post-treatment vs pre-treatment levels.

NI: not induced; I, induced

Figure S1: Low cytotoxicity of drugs at clinically relevant concentrations in leukaemic cell models assessed by Annexin V and propidium iodide (PI) staining.

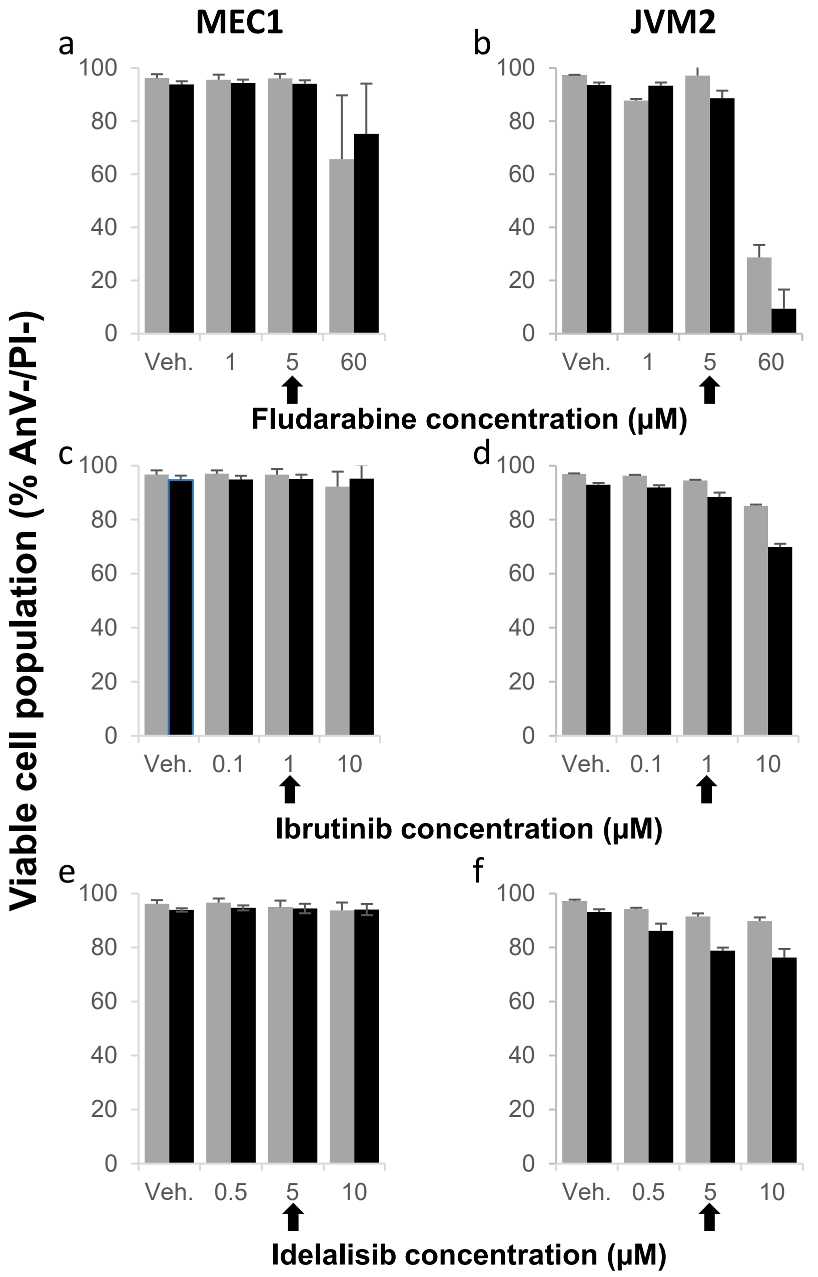

MEC1 (a, c, e) and JVM2 (b, d, f) cells expressing low or high UGT2B17 levels were exposed to increasing drug concentrations. Cells were stained with Annexin V (AnV) and propidium iodide (PI) 72 hours after treatment initiation. The AV-/PI- cells constituted the viable cell population. The cytotoxicity of fludarabine (a-b) was observed at high fludarabine concentrations in MEC1 (>40 uM) whereas fludarabine concentration above clinically relevant doses (>5 uM) were cytotoxic in JVM2. Ibrutinib (c-d) and idelalisib (e-f) displayed low cytotoxicity at clinically relevant concentrations (⬆).

Figure S2: Quantification of major *UGT1A* mRNAs expressed in (A) MEC1, (B) JVM2, and (C) EHEB lymphoid cells, and their induction by drug treatments.

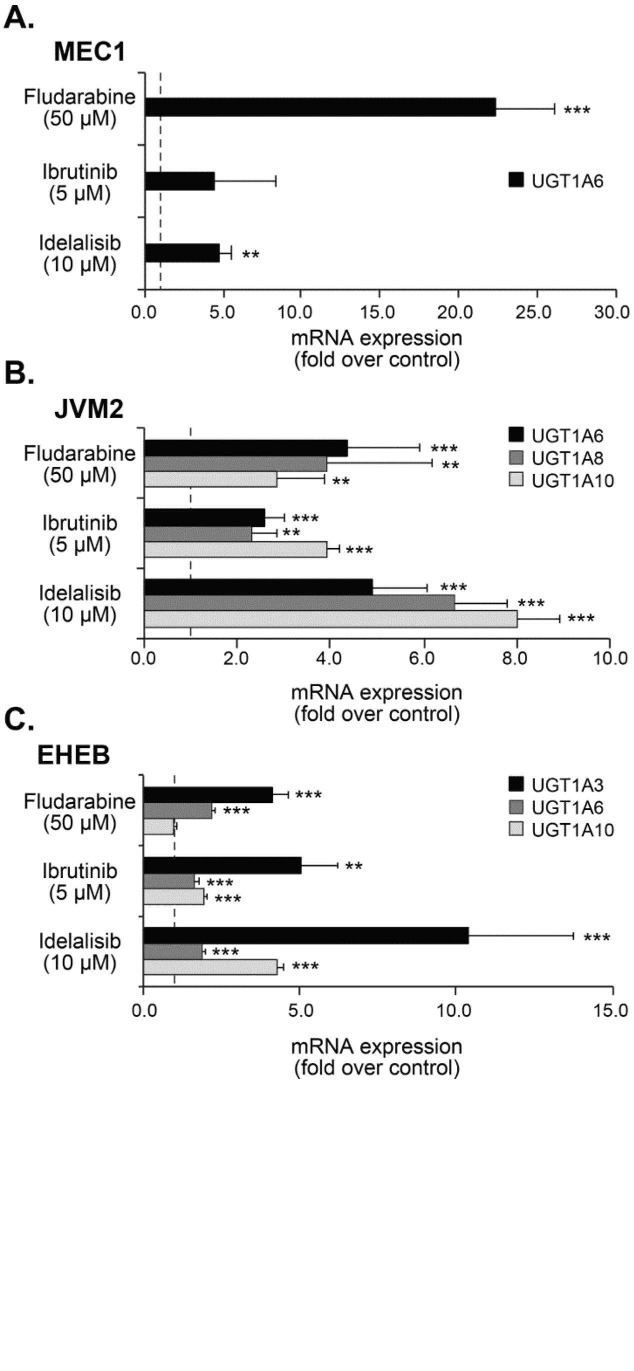

Figure S3: Complete enzymatic hydrolysis of fludarabine glucuronide conjugates G1 and G2 with β-glucuronidase demonstrates the formation of glucuronidated derivatives of fludarabine.

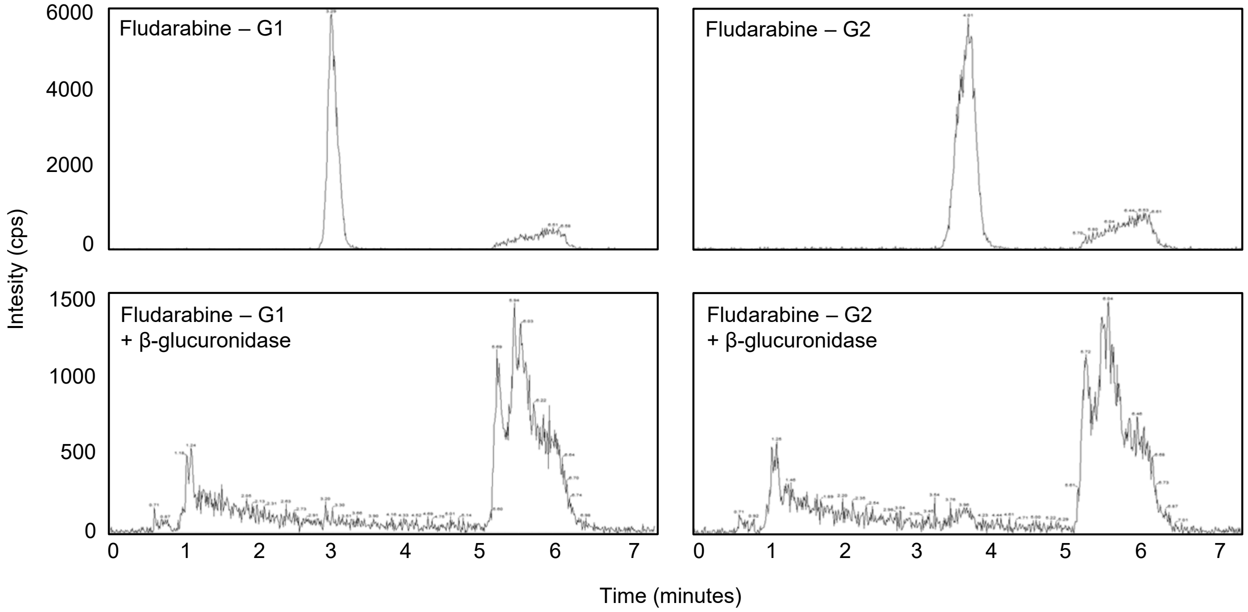

Supplement: Supplementary file 1 — Supplementary information [file 41416_2020_887_MOESM1_ESM.pdf]
